# Structural integrity of the anterior mid-cingulate cortex contributes to resilience to delirium in SuperAging

**DOI:** 10.1093/braincomms/fcac163

**Published:** 2022-06-28

**Authors:** Yuta Katsumi, Bonnie Wong, Michele Cavallari, Tamara G Fong, David C Alsop, Joseph M Andreano, Nicole Carvalho, Michael Brickhouse, Richard Jones, Towia A Libermann, Edward R Marcantonio, Eva Schmitt, Mouhsin M Shafi, Alvaro Pascual-Leone, Thomas Travison, Lisa Feldman Barrett, Sharon K Inouye, Bradford C Dickerson, Alexandra Touroutoglou

**Affiliations:** Harvard Medical School, Boston MA, USA; Frontotemporal Disorders Unit, Massachusetts General Hospital, Boston MA, USA; Department of Neurology, Massachusetts General Hospital, Boston MA, USA; Harvard Medical School, Boston MA, USA; Frontotemporal Disorders Unit, Massachusetts General Hospital, Boston MA, USA; Department of Psychiatry, Massachusetts General Hospital, Boston MA, USA; Harvard Medical School, Boston MA, USA; Center for Neurologlical Imaging, Department of Radiology, Brigham and Women’s Hospital, Boston MA, USA; Harvard Medical School, Boston MA, USA; Aging Brain Center, Marcus Institute for Aging Research, Hebrew SeniorLife, Boston MA, USA; Department of Neurology, Beth Israel Deaconess Medical Center, Boston MA, USA; Harvard Medical School, Boston MA, USA; Department of Medicine, Beth Israel Deaconess Medical Center, Boston MA, USA; Harvard Medical School, Boston MA, USA; Department of Psychiatry, Massachusetts General Hospital, Boston MA, USA; Frontotemporal Disorders Unit, Massachusetts General Hospital, Boston MA, USA; Department of Neurology, Massachusetts General Hospital, Boston MA, USA; Frontotemporal Disorders Unit, Massachusetts General Hospital, Boston MA, USA; Department of Neurology, Massachusetts General Hospital, Boston MA, USA; Department of Psychiatry and Human Behavior and Neurology, Brown University Warren Alpert Medical School, Providence RI, USA; Harvard Medical School, Boston MA, USA; Genomics, Proteomics, Bioinformatics and Systems Biology Center, Beth Israel Deaconess Medical Center, Boston MA, USA; Harvard Medical School, Boston MA, USA; Department of Medicine, Beth Israel Deaconess Medical Center, Boston MA, USA; Harvard Medical School, Boston MA, USA; Aging Brain Center, Marcus Institute for Aging Research, Hebrew SeniorLife, Boston MA, USA; Harvard Medical School, Boston MA, USA; Department of Neurology, Beth Israel Deaconess Medical Center, Boston MA, USA; Berenson-Allen Center for Non-Invasive Brain Stimulation, Beth Israel Deaconess Medical Center, Boston MA, USA; Harvard Medical School, Boston MA, USA; Berenson-Allen Center for Non-Invasive Brain Stimulation, Beth Israel Deaconess Medical Center, Boston MA, USA; Harvard Medical School, Boston MA, USA; Aging Brain Center, Marcus Institute for Aging Research, Hebrew SeniorLife, Boston MA, USA; Harvard Medical School, Boston MA, USA; Department of Psychiatry, Massachusetts General Hospital, Boston MA, USA; Department of Psychology, Northeastern University, Boston MA, USA; Harvard Medical School, Boston MA, USA; Aging Brain Center, Marcus Institute for Aging Research, Hebrew SeniorLife, Boston MA, USA; Department of Medicine, Beth Israel Deaconess Medical Center, Boston MA, USA; Harvard Medical School, Boston MA, USA; Frontotemporal Disorders Unit, Massachusetts General Hospital, Boston MA, USA; Department of Neurology, Massachusetts General Hospital, Boston MA, USA; Department of Psychiatry, Massachusetts General Hospital, Boston MA, USA; Massachusetts Alzheimer’s Disease Research Center, Massachusetts General Hospital, Boston MA, USA; Harvard Medical School, Boston MA, USA; Frontotemporal Disorders Unit, Massachusetts General Hospital, Boston MA, USA; Department of Neurology, Massachusetts General Hospital, Boston MA, USA

**Keywords:** successful aging, postoperative delirium, salience network, cortical thickness, MRI

## Abstract

Despite its devastating clinical and societal impact, approaches to treat delirium in older adults remain elusive, making it important to identify factors that may confer resilience to this syndrome. Here, we investigated a cohort of 93 cognitively normal older patients undergoing elective surgery recruited as part of the Successful Aging after Elective Surgery study. Each participant was classified either as a SuperAger (*n* = 19) or typically aging older adult (*n* = 74) based on neuropsychological criteria, where the former was defined as those older adults whose memory function rivals that of young adults. We compared these subgroups to examine the role of preoperative memory function in the incidence and severity of postoperative delirium. We additionally investigated the association between indices of postoperative delirium symptoms and cortical thickness in functional networks implicated in SuperAging based on structural magnetic resonance imaging data that were collected preoperatively. We found that SuperAging confers the real-world benefit of resilience to delirium, as shown by lower (i.e. zero) incidence of postoperative delirium and decreased severity scores compared with typical older adults. Furthermore, greater baseline cortical thickness of the anterior mid-cingulate cortex—a key node of the brain’s salience network that is also consistently implicated in SuperAging—predicted lower postoperative delirium severity scores in all patients. Taken together, these findings suggest that baseline memory function in older adults may be a useful predictor of postoperative delirium risk and severity and that superior memory function may contribute to resilience to delirium. In particular, the integrity of the anterior mid-cingulate cortex may be a potential biomarker of resilience to delirium, pointing to this region as a potential target for preventive or therapeutic interventions designed to mitigate the risk or consequences of developing this prevalent clinical syndrome.

## Teaser

Katsumi *et al*. report that, in older surgical patients, verbal recall performance comparable to young adults (‘SuperAging’) and greater cortical thickness of the anterior mid-cingulate cortex at baseline are associated with decreased severity of postoperative delirium. These results suggest the possible role of superior memory function in resilience to delirium.

## Graphical Abstract

**Graphical abstract:**
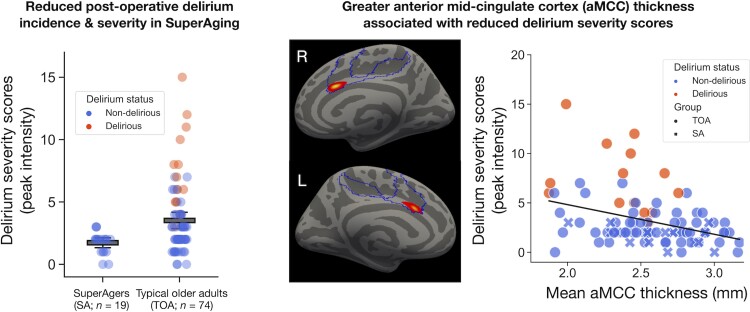

## Introduction

Delirium is an acute state of confusion marked by fluctuating symptoms of disturbance in attention, other cognitive functions, and often arousal.^1^ Postoperative delirium occurs in 11% to 51% of hospitalized older (age 65+) patients^2^ and substantially increases their risk for death, institutionalization, and dementia.^3^ Mortality rates among hospitalized patients who develop delirium are as high as those among patients with myocardial infarction or sepsis.^4^ Delirium costs more than $164 billion per year in the United States for all hospitalized older adults^5^ and more than $33 billion per year for older surgical patients,^6^ rivalling the costs associated with cardiovascular disease and diabetes. Despite its devastating clinical and societal impact, approaches to effectively treat delirium remain elusive. Major predisposing and precipitating factors of delirium include medical conditions that perturb brain homeostasis by altering cerebral structure and/or function, such as dementia, cerebrovascular disease, depression, infections, and psychoactive drugs.^1,7^ In addition to minimizing these risk factors, it is equally important to identify factors that may confer resilience to delirium. Clarification of a potential biomarker associated with resilience to delirium would be useful for appropriate targeting of interventions for this syndrome (e.g. via structural or functional neural plasticity) and to minimize its associated burden of downstream complications.

The pathogenesis of delirium is complex and likely reflects multiple aetiologies based on both underlying predispositions and superimposed acute stressors.^2,8^ In high-risk individuals, delirium is thought to represent a failure of the brain to show resilience to an acute stressor; this vulnerability is thought to be associated with a multitude of processes, including age-related impairment in brain network connectivity and neurodegeneration.^1^ Consistent with this view, accumulating evidence suggests that several different sets of interacting biological factors result in disruption of large-scale neuronal networks in the brain, leading to acute dysfunction in cognitive processes including attention, working memory, and executive functions.^9^ A recent meta-analysis of neuroimaging studies suggests that delirium is associated with pre-existing differences in some elements of brain structure and function, including local or regional atrophy, as well as structural and functional hypoconnectivity.^10^

A network-based model of delirium posits that the convergence of neurotransmitter changes, as well as neuroendocrine and inflammatory stressors on functional brain networks disrupts bottom-up and top-down attentional control, with the dysfunction of the salience network being a critical aspect of inattention in delirium.^11^ The salience network has been implicated broadly in the orienting of attention, motivation, and visceromotor regulation.^12–15^ In particular, the anterior mid-cingulate cortex (aMCC) is thought to be critical for tenacity (persistence in the face of challenge), likely owing to its role as a hub for domain-general signal integration and network coordination in the brain.^16^ This suggests the possibility that the integrity of the salience network—especially that of the aMCC—may be a critical component that supports resilience to delirium in older adults.

Consistent with this hypothesis, emerging research demonstrates that structural and functional preservation of the aMCC may be key to successful cognitive aging. Some investigators have focused on a remarkable subgroup of older adults, called ‘SuperAgers’,^17^ whose performance on some measures of episodic memory are statistically indistinguishable from those of middle-aged adults^17–20^ or even young adults,^21–23^ despite their advanced age. Many of the most pronounced neurobiological differences between SuperAgers (SAs) and typically aging older adults involve the structure and function of the aMCC. Anatomically, we and others have shown, based on structural MRI data, that the size of the aMCC in SAs is greater than typical older adults (TOAs).^17,19,20,22,24^ More recently, we further demonstrated with functional MRI data collected at wakeful rest that SAs also exhibited stronger intrinsic functional connectivity between the aMCC and other major nodes of the salience network when compared with TOAs.^23^ It is unclear exactly why the structural and functional integrity of the aMCC and other nodes of the salience network and many other networks decline in normal aging.^25^ However, the relatively few individuals who do not undergo these declines seem to exhibit memory performance comparable with that of young adults.

To the best of our knowledge, no study to date has investigated whether the preservation of key nodes of functional networks such as the salience network may confer resilience to delirium in older adults. Here, we sought to fill this gap by investigating the association between preoperative cortical thickness and the incidence and severity of postoperative delirium in a cohort of cognitively normal older patients undergoing elective surgery (for a detailed description of the study design and protocol, see Schmitt *et al*.^26,27^). Following closely the criteria used in previous studies of SuperAging,^17,20,22^ we identified SAs and TOAs within this cohort based on neuropsychological measures collected preoperatively at baseline. Based on the rationale described above, we hypothesized that (i) SAs would show reduced incidence and severity of postoperative delirium compared with TOAs, and that (ii) greater cortical thickness in the aMCC and other regions within the salience network at preoperative baseline would predict reduced incidence and severity of postoperative delirium in all participants.

## Methods

### Participants

**Figure 1:**
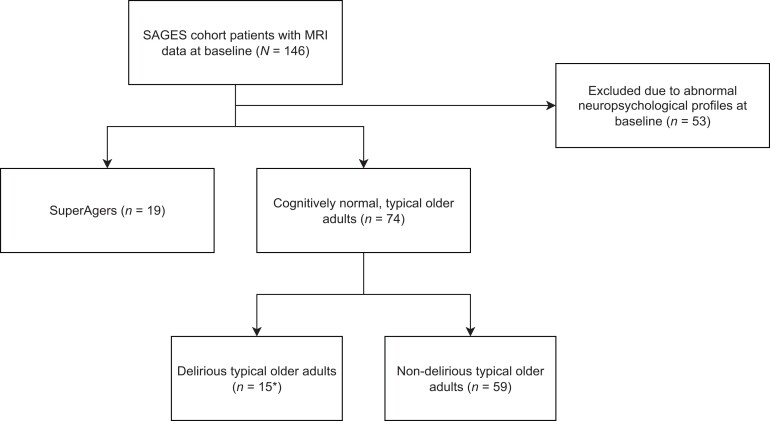
**Flow chart outlining the process of participant selection.** *One delirious typical older adult was excluded from all analyses involving CAM-S scores due to extreme values (*SD* > 3). All SA participants (*n* = 19) were postoperatively non-delirious.

Participants in this study were selected from the Successful Aging after Elective Surgery (SAGES) study. The SAGES study design and methods have been described in detail previously.^26,27^ Briefly, eligible participants were age 70 years and older, English speaking, scheduled to undergo elective surgery at one of two Harvard-affiliated academic medical centres, with an anticipated length of stay of at least 3 days. Eligible surgical procedures included: total hip or knee replacement; lumbar, cervical, or sacral laminectomy; lower extremity arterial bypass; open abdominal aortic aneurysm repair; and open or laparoscopic colectomy. Exclusion criteria included evidence of dementia, delirium, or hospitalization within 3 months prior to study participation, diagnosis of a terminal condition, legal blindness, severe deafness, history of schizophrenia or psychosis, and history of alcohol abuse or withdrawal. A total of 560 patients met eligibility criteria and were enrolled between 18 June 2010 and 8 August 2013. Written informed consent for study participation was obtained from all participants according to procedures approved by the institutional review boards of the two study hospitals [Beth Israel Deaconess Medical Center (BIDMC) and Brigham and Women’s Hospital], and the coordinating centre (Hebrew SeniorLife). In this study, we examined MRI and neuropsychological data acquired from the pool of 146 participants who had these data collected preoperatively and completed at least 6 months of postoperative follow-up.^28^ Of this initial pool of patients, 53 were excluded due to abnormal neuropsychological profiles at baseline (see **Results** and Fig. 1 below). This resulted in 93 patients (mean age: 75.8 ± 4.1; 57F/36 M), each of whom was classified as either a SA or TOA based on established neuropsychological criteria (see *SuperAging definition* below). One participant was excluded from all analyses involving Confusion Assessment Method-Severity (CAM-S) scores due to extreme values (*SD* >3).

### Neuropsychological testing

All participants completed an in-person battery of neuropsychological tests in their homes within 30 days before surgery [median, 9 days; interquartile range (IQR): 5–17]^29^ that included the following: Hopkins Verbal Learning Test-Revised (HVLT-R);^30^ Trail Making Test Parts A & B;^31^ digit span forwards and backwards (WAIS-III);^32^ Controlled Oral Word Association Test (F/A/S);^33^ Supermarket items;^34^ Boston Naming Test-15 (BNT-15);^35^ digit-symbol substitution test from the Repeatable Battery for the Assessment of Neuropsychological Status;^36^ and Visual Search and Attention Test.^37^ A Global Cognitive Performance (GCP) weighted composite was calculated for each participant reflecting general neuropsychological function encompassing performance on the entire battery administered and calibrated to the U.S. population.^38^

### SuperAging definition

We classified each participant as a SA or a TOA cross-sectionally on the basis of their neuropsychological test profiles, following prior work on SuperAging.^19–24,39–41^ In addition to normal performance according to published normative data on all neuropsychological tests administered, SAs were required to meet two additional psychometric criteria similar to those used in previous studies.^17,22^ Specifically, participants were required to perform at or above the mean gender-adjusted value for young adults (age range: 16–29 years old) on the Long Delay Free Recall trial of the HVLT-R. Participants were also required to perform no lower than 1 *SD* below the mean for their age and education group on the Trail Making Test Part B.^42^ All other participants not meeting these criteria were considered to be typically aging, cognitively normal older adults, provided their performance on all neuropsychological measures were within 1.5 *SD*s of published normative values for each neuropsychological instrument on the basis of age and education.

### Assessment of delirium

Delirium incidence and severity scores were assessed using a structured battery on each postoperative day throughout hospitalization. Delirium incidence was diagnosed using the Confusion Assessment Method (CAM)^43,44^ diagnostic algorithm, supplemented with a validated chart review method^45^ to detect the presence or absence of delirium for each patient. The CAM was rated based on information from patient interviews performed once daily in the late morning or early afternoon at approximately the same time each day; these included a brief cognitive screen (orientation, short-term recall, and sustained attention), the Delirium Symptom Interview,^46^ and information related to acute changes in mental status noted by nurses or family members.^26^ Study interviewers underwent intensive training and standardization.^27^ The CAM has high sensitivity (94%) and specificity (89%) for delirium^44^ and high inter-rater reliability (kappa statistic = 0.92 in 71 paired ratings in SAGES).^29^ The chart-based delirium instrument has a sensitivity of 74% and specificity of 83%.^45^ The CAM plus chart combined approach is the preferred method for detecting delirium since it maximizes sensitivity. Although the CAM detects the majority of delirium cases, the additional chart review increases sensitivity by identifying delirium throughout the 24 h period.^47^

Delirium severity scores were calculated using the CAM-S long form, which is based on the 10 features from the full CAM instrument to quantify the intensity of delirium features.^7^ CAM-S demonstrates strong psychometric properties and strong associations with important clinical outcomes.^7^ When inter-rater reliability for CAM-S long form in the SAGES data was evaluated in 73 pairs, the overall agreement was 97% and intraclass correlation coefficient was 0.88.^7^ Scores on the CAM-S long form range from 0 to 19, with higher scores indicating more severe delirium. Delirium severity scores were measured in the present study for each patient using both CAM-S peak (the highest single CAM-S rating observed during hospitalization) and CAM-S sum (the summed score across all hospital days), thereby capturing both delirium intensity and duration.^48,49^ It is important to note that CAM-S scores may be positive (>0) even in the absence of meeting full delirium criteria by CAM, reflecting either subsyndromal delirium or symptoms related to other conditions (such as dementia).

### MRI data acquisition and processing

We analyzed the magnetization-prepared fast gradient-echo (MPRAGE) 3D anatomical T_1_-weighted images (TR: 7.9 ms, TE: 3.2 ms, 15° flip angle, 32 kHz bandwidth, 24 × 19 cm field of view, 0.94 mm in-coronal plane resolution, 1.4 mm slices, preparation time of 1100 ms with repeated saturation at the beginning of the saturation period, and an adiabatic inversion pulse 500 ms before imaging) collected at the BIDMC Radiology Department on a 3 T HDxt MRI (General Electric Medical Systems) scanner using an 8-channel head coil.^28^ Each participant’s MPRAGE data underwent intensity normalization, skull stripping, and an automated segmentation of cerebral white matter to locate the grey–white boundary via FreeSurfer v6.0, which is documented and freely available online for download (http://surfer.nmr.mgh.harvard.edu). Defects in the surface topology were corrected,^50^ and the grey/white boundary was deformed outward using an algorithm designed to obtain an explicit representation of the pial surface. Each participant’s cortical surface reconstruction in their native space was registered to a template surface space (fsaverage) for intersubject comparisons.

### Statistical analysis

We performed a series of independent sample *t*-tests to statistically compare neuropsychological test performance between patients classified as SAs and TOAs, following previous studies of SuperAging. Similar analyses were performed to compare CAM-S sum and CAM-S peak scores between the two groups. The association between SA status and delirium incidence was assessed via a Chi-square test of independence. This analysis revealed that all of the patients who developed delirium postoperatively were TOAs (see **Results**). Therefore, we performed a series of one-way analysis of variance (ANOVA) to compare neuropsychological performance across the three groups: SAs, postoperatively delirious TOAs (D-TOAs), and non-delirious TOAs (ND-TOAs). Turning to the analysis of cortical thickness data, to identify regions of the cerebral cortex whose thickness is associated with delirium incidence and severity across all participants, we created a vertex-wise general linear model (GLM) in FreeSurfer. Given our a priori hypotheses regarding the salience network, we restricted our analysis within the boundaries of this network using an established parcellation of the cerebral cortex (the ‘ventral attention’ network; Yeo *et al*.^51^). Statistical significance was assessed using an uncorrected vertex-wise threshold of *P* < 0.05 within this network mask, following similar approaches employed in prior work on SuperAging.^22,23^ This analysis revealed areas of the cerebral cortex where cortical thickness was associated with CAM-S sum and/or peak scores across all patients regardless of postoperative delirium status. To ensure the specificity of our results, we also performed a whole-cortex GLM analysis without any network masks. Finally, we conducted a one-way ANOVA, as well as *t*-tests to perform a *post hoc* group comparison of the mean aMCC cortical thickness based on the significant clusters identified from our vertex-wise analysis described above. Analyses were conducted using the *mri_glmfit* function in FreeSurfer, with additional group-level statistical tests performed using SPSS Statistics v27 (IBM Corp.); statistical significance was assessed at *P* < 0.05.

### Data availability

The data that support the findings of this study are available from the corresponding authors, upon reasonable request.

## Results

Figure 1 describes the process of participant selection. Of the initial pool of 146 patients, 53 of them were excluded due to scoring 1.5 *SD* or lower relative to the published normative value for at least one of the neuropsychological instruments adjusted for age and education. Demographic and neuropsychological characteristics of the remaining 93 patients in our study sample are summarized below in Table 1. Nineteen (20%) of the 93 patients were classified as SAs based on their performance on the HVLT-R delayed recall measure and Trail Making Test Part B, relative to the gender-adjusted mean value for young adults (see Methods), while the remaining 74 patients were considered TOAs. All TOAs performed within the normative values for their age and sex on at least the HVLT-R and Trail Making Test Part B, if not across all other neuropsychological tests administered as part of the battery. In addition to the HVLT-R and Trail Making Test Part B, SAs performed better on measures of processing speed, most tests of executive functions, and other measures of episodic memory. We found no statistically significant differences between groups in age (*P* ≤ 0.71), education (*P* ≤ 0.28), or the distribution of sexes (*P* ≤ 0.21).

**Table 1:** Demographic and neuropsychological characteristics of SAs and TOAs

Neuropsychological measure	SuperAger	Typical older adult
*n*	19	74
Sex (% female)	74	58
Age (years)	75.5 (4.5)	75.9 (4.0)
Education (years)	16.1 (3.0)	15.2 (2.7)
Trail Making Test A (*s*)	31.3 (6.9)**	36.9 (10.6)
**Trail Making Test B (*s*)**	**62.1** (14.0)**	**100.8** **(38.3)**
HVLT-R Trial 1 (12)	7.9 (1.9)**	6.1 (1.4)
HVLT-R Trial 3 (12)	11.4 (0.8)**	9.4 (1.6)
HVLT-R total learning (36)	29.7 (3.2)**	23.9 (4.0)
**HVLT-II Delayed Recall (12)**	**11.4** (0.5)**	**8.1** **(1.5)**
HVLT-R % retention [(Delayed recall/Higher score of Trials 2 and 3) x 100]	99.9 (7.3)**	85.0 (12.5)
HVLT-R Recognition (true positives)	11.9 (0.3)**	11.3 (0.9)
HVLT-R Recognition Discrimination Index (Total true positives – total false positive)	11.4 (0.8)**	10.6 (1.3)
VSAT (# correct)	47.1 (10.4)	47.9 (8.8)
Digit span forward	7.1 (1.4)	6.6 (1.2)
Digit span backward	5.5 (1.4)*	4.8 (1.1)
Digit-symbol substitution (# correct)	44.4 (7.4)**	38.5 (9.1)
Controlled Oral Word Association Test (F/A/S)	46.2 (8.7)**	38.1 (10.5)
Category fluency (supermarket items)	26.9 (5.6)*	23.5 (5.3)
Boston Naming Test-15	14.5 (1.0)	13.9 (1.3)
MMSE	27.6 (0.8)*	26.5 (1.2)
GCP	66.5 (4.6)*	59.8 (5.1)

Values represent means and standard deviations (in parentheses) for each neuropsychological measure. HVLT total learning = sum of word recalled across all encoding trials. Values in parentheses in the leftmost column indicate maximum score unless otherwise specified. Bolded measures were used to classify each patient as either a SA or TOA. s = seconds; *SD* = standard deviation. Asterisks denote statistically significant differences from TOAs at *P ≤ 0.05 or **P ≤ 0.01.

### Incidence and severity of postoperative delirium between SuperAgers and typical older adults

**Figure 2:**
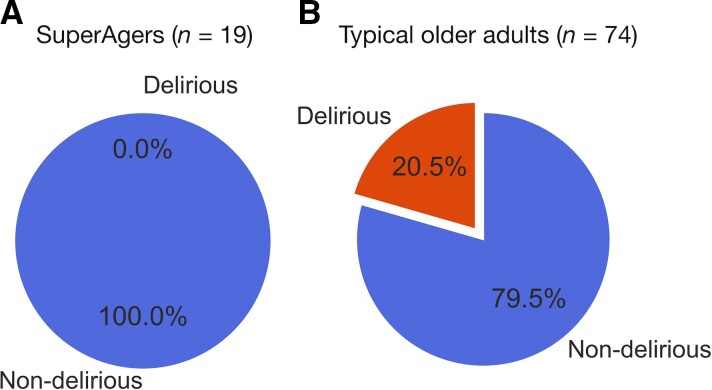
**Incidence of postoperative delirium** in (**A**) SAs versus (**B**) TOAs.

**Figure 3:**
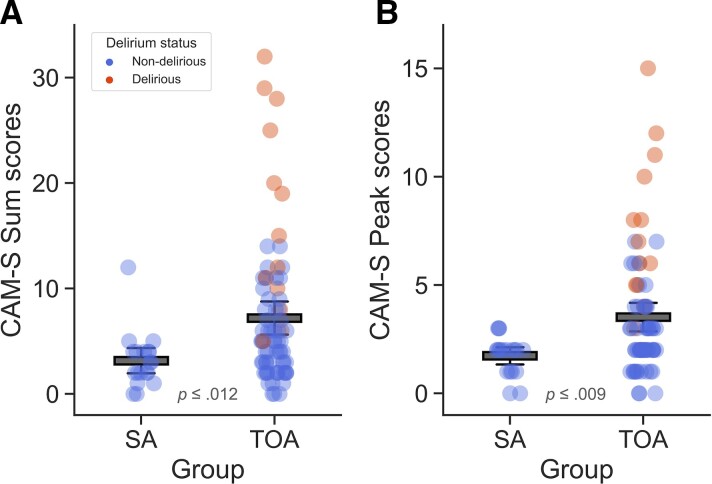
**Postoperative delirium severity by group.** Severity scores of postoperative delirium in SAs versus TOAs expressed as (**A**) intensity × duration and (**B**) highest intensity. *P*-values are associated with paired samples *t*-tests. Error bars denote 95% confidence intervals. Coloured circles represent individual subjects in the sample. SA = SuperAgers, TOAs = typical older adults.

A χ^2^ test of independence revealed a significant association between SuperAger status and delirium incidence: *x*^2^ ≥ 4.59, *P* ≤ 0.032. Specifically, 15 patients developed delirium postoperatively during hospitalization, all of whom were TOAs (Fig. 2). Neuropsychological characteristics of SAs, postoperatively D-TOAs, and ND-TOAs are summarized in Table 2. Overall, SAs performed better than both D-TOAs and ND-TOAs in most aspects of episodic memory function as measured by the HVLT-R. Turning to delirium severity scores, we found that SAs on average showed lower CAM-S ratings than TOAs, both in terms of CAM-S sum (intensity × duration) (SAs: *M* = 3.16, *SD* = 2.63 versus TOAs: *M* = 7.21, *SD* = 6.75; *t*(90) = 2.56, *P* ≤ 0.012) and peak (highest intensity) (SAs: *M* = 1.74, *SD* = 0.87 versus TOAs: *M* = 3.52, *SD* = 2.82; *t*(90) = 2.71, *P* ≤ 0.009) scores (Fig. 3).

**Table 2:** Demographic and neuropsychological characteristics of SAs, postoperatively D-TOAs and ND-TOAs

Neuropsychological measure	SuperAger (SA)	Delirious (D) typical older adults	Non-delirious (ND) typical older adults	Group differences
*N*	19	15	59	-
Sex (% female)	74	60	58	-
Age (years)	75.5 (4.5)	75.7 (4.3)	76.0 (4.0)	-
Education (years)	16.1 (3.0)	14.9 (2.5)	15.3 (2.7)	-
Trail Making Test A (s)	31.3 (6.9)	38.1 (10.7)	36.6 (10.7)	SA < ND^a^
**Trail Making Test B (s)**	**62.1** (**14.0)**	**106.1** (**47.1)**	**99.4** (**36.1)**	SA < ND = D^b^
HVLT-R Trial 1 (12)	7.9 (1.9)	6.1 (1.3)	6.2 (1.4)	SA > ND = D^b^
HVLT-R Trial 3 (12)	11.4 (0.8)	9.3 (1.3)	9.4 (1.6)	SA > ND = D^b^
HVLT-R total learning (36)	29.7 (3.2)	23.9 (3.3)	23.9 (4.1)	SA > ND = D^b^
**HVLT-II Delayed Recall (12)**	**11.4** (**0.5)**	**8.1** (**1.4)**	**8.1** (**1.5)**	SA > ND = D^b^
HVLT-R % retention [(Delayed recall/Higher score of Trials 2 and 3) x 100]	99.9 (7.3)	85.1 (11.9)	85.0 (12.7)	SA > ND = D^b^
HVLT-R Recognition (true positives)	11.9 (0.3)	11.3 (0.8)	11.2 (0.9)	SA > ND^b^; SA > D^c^
HVLT-R Recognition Discrimination Index (Total true positives – total false positive)	11.4 (0.8)	10.7 (0.9)	10.5 (1.4)	SA > ND^b^
VSAT (# correct)	47.1 (10.4)	51.1 (7.9)	47.0 (8.9)	-
Digit span forward	7.1 (1.4)	6.7 (1.3)	6.6 (1.2)	-
Digit span backward	5.5 (1.4)	5.0 (1.2)	4.7 (1.1)	-
Digit-symbol substitution (# correct)	44.4 (7.4)	43.4 (8.4)	37.3 (8.9)	D > ND^a^
Controlled Oral Word Association Test (F/A/S)	46.2 (8.7)	40.0 (10.2)	37.7 (10.6)	SA > ND^b^
Category fluency (supermarket items)	26.9 (5.6)	24.4 (4.6)	23.3 (5.5)	SA > ND^a^
Boston Naming Test-15	14.5 (1.0)	14.1 (1.6)	13.9 (1.2)	-
MMSE^d^	27.6 (0.8)	26.7 (1.1)	26.5 (1.3)	SA > ND^b^; SA > D^a^
GCP	66.5 (4.6)	61.7 (5.2)	59.3 (5.9)	SA > ND^b^; SA > D^a^

Values represent means and standard deviations (in parentheses) for each neuropsychological measure. HVLT total learning = sum of word recalled across all encoding trials. Values in parentheses in the leftmost column indicate maximum score unless otherwise specified. Bolded measures were used to classify each patient as either a SA or TOA. s = seconds; *SD* = standard deviation.

^a^ *P* ≤ 0.05

^b^ *P* ≤ 0.01

^c^ *P* ≤ 0.054

^d^ MMSE score calculated from 3MS measured at baseline.

### Greater baseline cortical thickness of the aMCC is associated with reduced postoperative delirium severity

**Figure 4:**
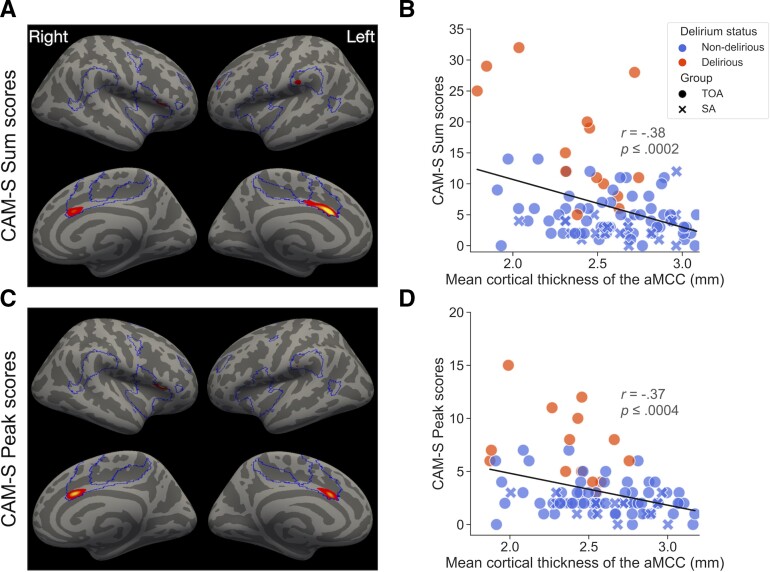
**Greater cortical thickness in the anterior mid-cingulate cortex at baseline is associated with reduced delirium severity scores following major elective surgery.** Coloured vertices on the cortical surface maps indicate areas within the salience network (represented with solid line borders), where cortical thickness was negatively associated with (**A**) CAM-S sum and (**C**) peak scores. Scatter plots on depict the relationship between mean cortical thickness extracted from bilateral aMCC clusters within the respective surface maps and (**B**) CAM-S sum and (**D**) peak scores. Coloured crosses (SAs) and circles (TOAs) represent individual participants. Spearman’s rank correlation coefficients revealed similarly significant associations for CAM-S Sum (*r* = -0.27, *P* ≤ 0.009) and peak (*r* = -0.33, *P* ≤ 0.0013) scores.

A vertex-wise GLM analysis revealed that the severity of postoperative delirium, as measured by CAM-S sum and peak scores, was negatively associated with the thickness of cortical regions within the salience network at baseline, including bilateral aMCC and smaller, unilateral clusters part of the dorsal anterior insula, anterior middle frontal gyrus, and the supramarginal gyrus (Fig. 4). An independent GLM in which sex, age, and GCP of each participant were included as covariates of no interest revealed very similar results (Supplementary Fig. 1). To assess the specificity of the observed effect to the salience network, we performed a similar GLM analysis but without any masking of the results. This whole-cortex analysis revealed few clusters outside the boundaries of the salience network, with bilateral aMCC still showing the most significant effect (Supplementary Fig. 2). These results suggest that the aMCC, a key node of the salience network, may play a uniquely protective role against postoperative delirium in surgical patients, consistent with its role in successful cognitive aging. In addition, we computed for each participant the mean cortical thickness of the significant aMCC clusters, and statistically compared it across SAs, D-TOAs and ND-TOAs. A one-way ANOVA revealed a trend-level effect: *F*(2,89) = 3.09, *P* ≤ 0.0503. *Post hoc* tests identified greater aMCC thickness in SAs (*M* = 2.62, *SD* = 0.27) than D-TOAs (*M* = 2.37, *SD* = 0.29) (*t*(31) = 2.47, *P* ≤ 0.019, Cohen’s *d* = 0.89), with no difference in thickness when compared with ND-TOAs (*M* = 2.59, *SD* = 0.32) (*t*(76) = 0.72, *P* ≤ 0.72, *d* = 0.10). Greater aMCC thickness was also observed for ND-TOAs compared with D-TOAs (*t*(71) = 2.26, *P* ≤ 0.27, *d* = 0.72) (Supplementary Fig. 3).

## Discussion

Because of the expected increase in the older population over the coming decades^52^ and a burgeoning number of older persons undergoing major surgery,^53,54^ substantial numbers of people are at elevated risk for developing postoperative delirium, as well as longer-term cognitive decline.^29,55^ Since various types of brain structural and functional decline play a prominent role in delirium pathophysiology,^1^ there is an urgent need to advance our understanding of the mechanistic underpinnings of resilience to delirium. Here, we show that SuperAging—so-defined using straightforward neuropsychological measures—confers the real-world benefit of resilience to delirium in older adults undergoing elective surgery: None of the SAs demonstrated full clinical delirium by CAM criteria. Furthermore, greater pre-surgical cortical thickness of the aMCC—a key node of the brain’s salience and frontoparietal networks reliably implicated in SuperAging—was associated with reduced postoperative delirium severity scores. This suggests that superior memory function in older adults^22–24^ may predict reduced risk and severity of postoperative delirium, possibly via a protective effect of the structural integrity of the aMCC. Given that the aMCC exhibits high neural plasticity and its structure and function can be improved with training,^56,57^ this brain region may be one potential ‘target’ biomarker to engage in intervention strategies aiming to prevent delirium.

A systematic review of published delirium risk prediction models revealed that cognitive impairment was second only to age as the most commonly replicated predictor of delirium.^58^ Consistent with this evidence, we previously showed that a baseline measure of general cognitive performance assessed preoperatively comprising a variety of domains (e.g. attention, executive function, memory, language, visuospatial processing) is the dominant predictor of delirium risk in a larger (*N* = 500+) sample of older adults.^59^ Similarly, another study showed that greater participation in cognitive activities (e.g. reading, writing, playing games) in general was associated with reduced incidence and severity of postoperative delirium in a sample of older adults undergoing elective orthopaedic surgery.^60^ Here, we extend these findings by demonstrating that, when undergoing elective surgery, older adults with verbal memory performance comparable with that of young adults are more likely to be protected against the development and prolongation of postoperative delirium. This suggests that it may be possible to select a succinct battery of tests that could be used in preoperative cognitive risk assessment. This is consistent with the growing literature demonstrating the utility of relatively brief and inexpensive cognitive testing in predicting the degree of adverse outcomes in hospitalized older patients.^61–63^

Consistent with prior results,^17,20,22–24^ we found that the aMCC is a key brain region relevant to successful aging. In the current sample, the older adults who were classified as SAs had greater cortical thickness in the aMCC than TOAs who developed delirium postoperatively. This group difference in aMCC thickness of ∼0.25 mm on average is consistent with the findings of prior work on SAs.^22,24^ Cortical thickness in the aMCC did not differ when SAs were compared with TOAs who did not develop delirium postoperatively, a finding that may be driven by a few factors. One possibility, for instance, is that TOAs who did not develop delirium postoperatively in the current sample were higher-functioning than TOAs examined in other studies. Consistent with this speculation, general cognitive performance in the SAGES sample as a whole has been shown to be on average 0.8 *SD*s above the population mean in the U.S.^26^ Relatively high cognitive function across domains, therefore, might be another factor associated with greater cortical thickness in the aMCC. This idea fits with meta-analytic observations that the aMCC and other regions of the salience network are consistently activated during a variety of cognitive tasks.^64^ This is also consistent with the current finding that SAs’ superior cognitive performance was not specific to delayed recall but was more general across multiple cognitive domains, as has been observed in prior studies of SuperAging.^19,24,39^ We also previously identified a similar pattern of associations across different neuropsychological measures in a sample of post-surgical patients.^38^ Alternatively, it is also possible that the lack of differences in aMCC thickness is driven by the unequal size of subsamples. In the present study, the majority of its participants were classified as ND-TOAs, with similar numbers of the remaining participants classified as SAs or D-TOAs. Higher precision estimates of brain structure could help shed light on this possibility.^65^ Nonetheless, our finding lends support to the growing body of evidence that the aMCC is an important region of the circuitry underlying successful aging.^16,19,20,22–24^

Our findings also revealed that structural integrity of the aMCC is an important predictor of postoperative delirium severity scores. Although delirium appears to be associated with differences in some features of brain structure (e.g. global/local atrophy, white matter hyperintensity) and function (e.g. reduced cerebral blood flow, alterations in functional connectivity), a recent meta-analysis identified mixed results.^10^ The mixed results may be due to the heterogeneous nature of delirium, as well as variability in study designs, imaging data modalities and analytical procedures. Prior work from our group showed that cortical thickness calculated in a specific set of regions known to exhibit atrophy in Alzheimer’s disease^66–69^ was associated with delirium incidence and severity.^70^ White matter degradation in related areas (e.g. frontal cortex, hippocampus) was also associated with delirium severity.^71^ In contrast, measures of global brain atrophy were not related to delirium, suggesting the need for more targeted approaches to specific networks or regions of interest.^28^

The role of the aMCC in resilience to delirium likely relates to its central role in signal integration in the brain, as well as regulation of the body’s internal systems. Owing to its position at the intersection of multiple intrinsic brain networks, the aMCC is a major hub that allows efficient functional communication across spatially distributed brain regions.^72–76^ It is therefore not surprising that its role has been implicated in various aspects of cognitive and affective functions, including attention, executive function, emotion, and motivation.^16^ Even more generally, the aMCC is a key component of brain systems responsible for issuing visceromotor control signals to maintain the body’s internal milieu (e.g. the autonomic nervous system, the immune system, the endocrine system) and to coordinate skeletomotor movements, all in the service of predictively managing the body’s energy resources.^13,77^ Therefore, structural integrity of the aMCC may be important for reducing the likelihood of developing symptoms including arousal and psychomotor disturbance and metabolic dysfunction, both of which have been shown to characterize subtypes of delirium.^78,79^ More research is needed to explicate the link between structural and functional properties of the aMCC and the pathophysiology of delirium.

A few limitations of this study are noteworthy. First, using a prospective cohort design, the present sample included disproportionately fewer postoperatively delirious patients than ND-TOA patients. The current sample also consisted of disproportionate numbers of SA and TOA participants, although the number of SAs was similar to those of previous studies.^22,24^ Future work with larger overall samples would be warranted, so that the observed effects can be tested with greater numbers of delirious cases and SAs. It would also be important to evaluate these effects with the implementation of correction for multiple statistical comparisons in future confirmatory studies. Second, while the internal validity of the study is not compromised, the generalizability of our findings may be limited, given that the current subject sample consisted of participants with a high mean educational level. Future studies should examine other populations including adults hospitalized for reasons other than surgery (or different types of surgery than those studied herein) and those who develop delirium unrelated to surgery. Greater diversity in geographical, cultural, and socioeconomic characteristics would also be warranted. Finally, despite our use of a well-validated approach for detection, we acknowledge that delirium is a fluctuating condition and any measurement approach may have false negatives, contributing to potential measurement error.

Notwithstanding these potential limitations, the new evidence identified in the present study sheds light on the possible neural mechanisms underlying resilience to postoperative delirium in aging by highlighting the key role of the aMCC. Future studies should look beyond this region by examining aspects of functional and structural connectivity of the aMCC and their potential association with delirium. This line of work is warranted by our prior findings showing that diffusion imaging biomarkers assessed at baseline are associated with postoperative delirium incidence and severity.^71^ A connectivity-based approach would be useful in clarifying the extent to which the effect of delirium is localized to the aMCC or can be better characterized with the involvement of a broader network of brain regions. In addition, although our findings did not appear to be influenced by the effect of sex, SAs in this sample clearly had a more dominant female presence (70+%), a pattern consistent with previous studies of SuperAging.^17,22,24^ Future neuroimaging studies might examine the interaction of SuperAging and sex, given that females on average exhibit greater cortical thickness across widespread regions of the cerebral cortex than males, whereas males exhibit greater volumes and surface areas than females.^80^ Furthermore, because we identified SAs cross-sectionally in this study, it remains unclear whether they have always showed higher performance than their typically aging counterparts across the lifespan (while showing cognitive decline at the same rate) or are more resistant or resilient to age-related cognitive decline. More work is needed to better characterize the trajectory of neural and cognitive changes in SAs, as current longitudinal evidence testing these possibilities is mixed.^18,24,39,81^

Future work should also examine the possibility of minimizing the risk of developing delirium through interventions targeting neural plasticity (both structurally and functionally) of the aMCC. There is evidence showing that protein receptors important for synaptic plasticity (e.g. CaMKII, NR2B, NMDA) are highly expressed in this region.^82–84^ These protein receptors are thought to be activated during long-term potentiation of synaptic strength that occurs with experience (e.g. learning) and to contribute to the subsequent enlargement and strengthening of the synapse.^85,86^ Furthermore, consistent with its role in coordinating the visceromotor and skeletomotor systems, the aMCC appears to be sensitive to aerobic exercise interventions in older adults, showing an increase in grey matter volume after 6 months of training^56,57^ with concomitant improvement in episodic memory function.^57^ These findings point to the role of the aMCC as one potential biomarker of resilience to delirium to be targeted by preventive or therapeutic intervention programmes designed to mitigate the risk or consequences of developing postoperative delirium. Such strategies might involve exercise interventions or non-invasive brain stimulation (e.g. repetitive transcranial magnetic stimulation) that has been shown to induce neuroplasticity through modulation of receptor expressions.^87^ Ultimately, we hope that this and other biomarkers related to risk for or resilience to delirium could be used to identify older adults for ‘pre-habilitation’ interventions to maximize their ability to tolerate the stresses of elective surgery or other medical treatments.

## Supplementary Material

fcac163_Supplementary_Data

## Abbreviations

aMCC =: anterior mid-cingulate cortex
CAM =: Confusion Assessment Method
D-TOA =: delirious typical older adult
GLM =: general linear model
MRI =: magnetic resonance imaging
ND-TOA =: non-delirious typical older adult
SA =: SuperAger
SAGES =: the Successful Aging after Elective Surgery study
TOA =: typical older adult
