# Supplementary material for: Structural integrity of the anterior mid-cingulate cortex contributes to resilience to delirium in SuperAging
Source: Brain Commun. 2022 Jun 28;4(4):fcac163. doi: 10.1093/braincomms/fcac163 (PMC9272062; doi:10.1093/braincomms/fcac163)
Supplement: fcac163_Supplementary_Data [file fcac163_supplementary_data.pdf]

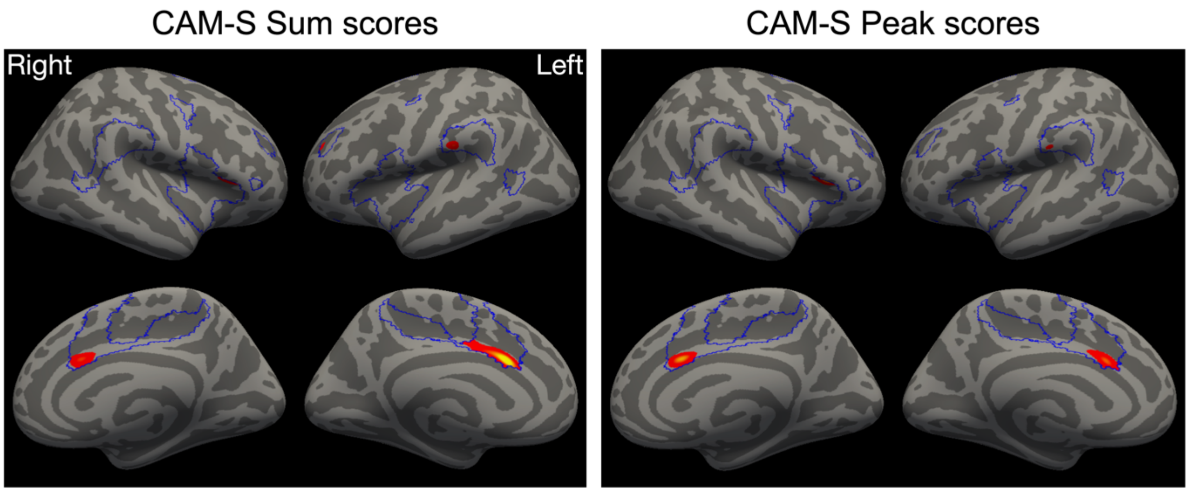

Supplementary Figure 1. Vertex-wise regression of delirium severity on cortical thickness controlling for age, sex, and GCP.

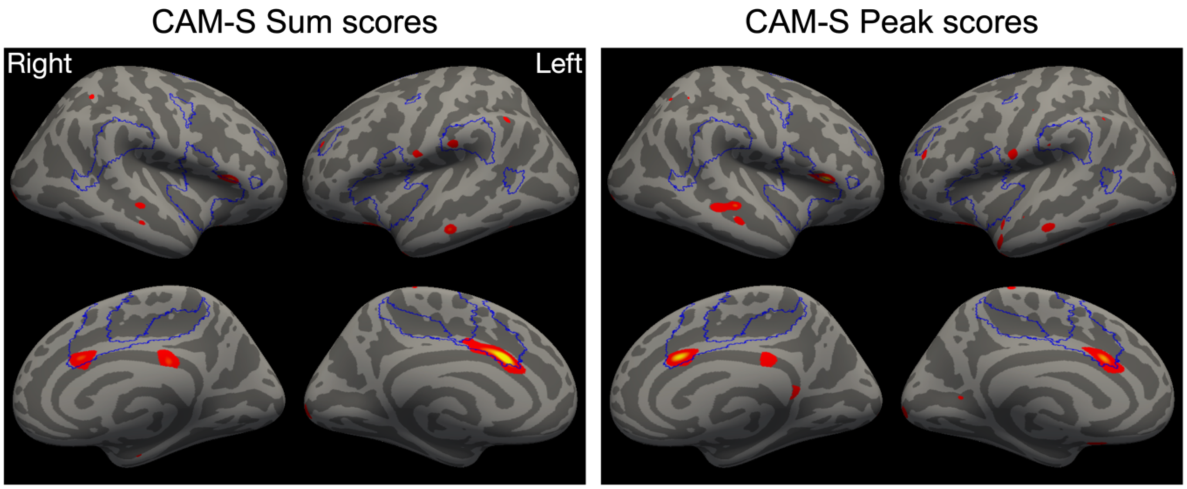

Supplementary Figure 2. Whole-cortex vertex-wise regression of delirium severity on cortical thickness. Analyses examining all cortical vertices yielded similar results to those shown in Figure 3, suggesting that cortical thickness outside the salience network was not strongly associated with measures of delirium severity.

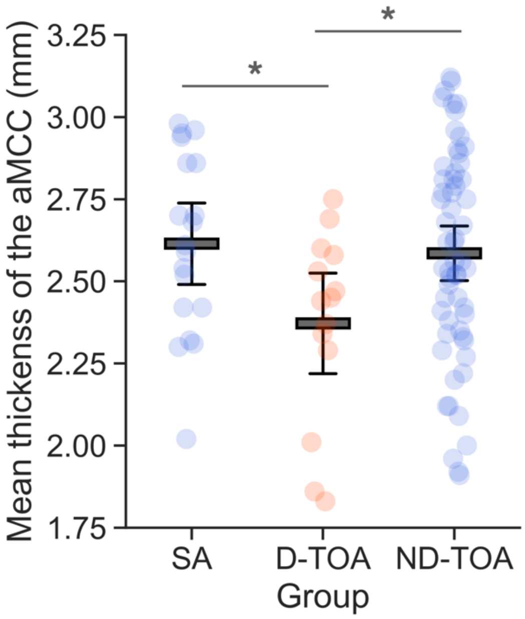

Supplementary Figure 3. Mean cortical thickness of the anterior mid-cingulate cortex showing significant associations with delirium severity scores. Horizontal bars indicate the mean cortical thickness of the anterior mid-cingulate cortex (aMCC) extracted for each group from bilateral clusters shown on the surface maps in **Figure 4** (see main text). Error bars denote 95% confidence intervals. Colored circles represent individual subjects in each group, the color of which is consistent with their status of post-operative delirium. SA = SuperAger; D-TOA = delirious typical older adult; ND-TOA = non-delirious typical older adult. *$p \leq .05$.
